# Control Group Selection in Preclinical Rat Bone Defect Models: A Systematic Review

**DOI:** 10.3390/jfb17020066

**Published:** 2026-01-28

**Authors:** Lotta Reimann, Emma Marchionatti, Adrian Steiner, Stephan Zeiter, Caroline Constant

**Affiliations:** 1 AO Research Institute Davos, 7270 Davos, Switzerland; lotta.reimann@t-online.de (L.R.); stephan.zeiter@aofoundation.org (S.Z.); 2 Vetsuisse-Faculty Bern, University of Bern, 3012 Bern, Switzerland; emma.marchionatti@uzh.ch (E.M.);; 3 Vetsuisse-Faculty Zurich, University of Zurich, 8057 Zurich, Switzerland

**Keywords:** biomaterials, bone graft, control groups, femur, bone, rats

## Abstract

Large bone defects and loss present major orthopedic challenges. In preclinical research, femoral bone defects in rats are commonly used as in vivo models to evaluate new osteoregenerative biomaterials. These test items are typically compared to negative and positive controls. This review aims to summarize the different control groups used to evaluate new osteoregenerative test items in preclinical rat femoral defect models and to identify potential pitfalls related to these controls, ultimately to enhance the future translational success. The protocol for this review was registered in PROSPERO, and no specific funding was received for this work. The systematic search comprised publications between January 2001 and January 2023. 436 studies were included for analysis. The choice of control groups was inconsistent across studies. A negative (e.g., empty defects or inert carriers) and positive (e.g., bone grafts or commercially available bone substitutes) control group was included in 56% (*n* = 245/436) and 34% (*n* = 149/436) of the included studies, respectively. Notably, 25% (*n* = 109/436) of the studies did not include any control group. Bone grafts were used as positive controls in 50% of the studies that included positive controls (*n* = 74/149), mainly of allogeneic origin (45%, *n* = 33/74). The control groups used to evaluate the test item impacted the healing comparison, with 81% of studies showing better healing of their test items compared to negative control (*n* = 198/245) versus 54% compared to positive control (*n* = 80/149). A qualitative risk-of-bias and reporting assessment was performed using an integrated ARRIVE–SYRCLE framework. Most studies demonstrated moderate concern in several domains, with frequent absence of randomization (67%, high concern) and blinding (84%, high concern), incomplete reporting of inclusion/exclusion criteria (74%, moderate concern), and variable clarity regarding animal characteristics and statistical methodology. The variability in the choice of control groups appears to influence study outcomes. Inadequate control group selection can lead to misleading conclusions regarding the efficacy of new biomaterials. Therefore, standardizing control group selection is crucial to enhance the reliability and comparability of preclinical research findings.

## 1. Introduction

Large bone defects and loss are among the most prominent orthopedic challenges that receive considerable attention in bone surgery [1]. Worldwide, 2 million bone defects alone are repaired with bone grafts yearly [2]. In many cases, they are essential to provide healing support and avoid prolonged disabilities or the need for amputation. To this aim, the current gold-standard treatment is the transplantation of an autologous bone graft [3,4], meaning that bone tissue is harvested from the same individual receiving the graft. In this situation, bone healing can be enhanced due to osteoconduction, osteoinduction, osteogenesis, and osteointegration properties, depending on the type of bone graft harvested [3,5,6,7]. Furthermore, autologous bone grafts carry a very low risk of postoperative foreign body reaction [5,6,8], due to their high histocompatibility [3]. However, autologous bone grafting is linked to several morbidities, including risks from extended general anesthesia, additional postoperative pain, and delayed healing at the donor site due to possible infection or hematoma formation [3,6,9,10,11]. Moreover, some patients may offer insufficient amount or quality of bone graft material to qualify for its practical application [10,11]. Therefore, there is a massive need to develop new, safe biomaterials for large bone defects capable of replacing autologous bone grafts.

Developing new biomaterials is a multistep procedure, with in vivo testing being a central aspect of assessing the safety and efficacy of treatments for bone defects. This testing is often performed in rodents, preferably rats, using femoral defect models [12,13,14]. Rats are frequently selected as the first preclinical in vivo model due to their small size, ease of handling, similarity to human bone in terms of anatomical and physiological properties, and the availability of extensive knowledge of bone healing from well-established defect models [12,14]. Adequate control groups are crucial in these studies, as they provide a basis for comparing test items (e.g., biomaterials) and conclude on their healing properties. Negative controls, such as empty bone defects, assess whether a test item can enhance bone healing compared to the natural process. On the contrary, positive controls are used to compare the healing properties of test items against proven gold standards in clinical practice, such as autologous bone grafts.

Failure to use appropriate control groups can lead to misleading conclusions, reducing the ability to translate findings into clinical and commercial applications, which may explain why so few artificial biomaterials have progressed to clinical use [15]. Standardizing the use of bone grafts as positive controls, including their origin (autologous, allogeneic, xenogeneic), could prevent misinterpretation, improve comparability across studies, and enhance the translational potential of biomaterials. Poorly designed studies waste animal lives, human trial participants, money, and time.

A review of preclinical bone defect models in rats and current research practices regarding control group selection for investigation of new biomaterials is essential, given the need to standardize research protocols and improve the translation ability of such studies to commercial use in humans. While previous narrative reviews have reported bone defect models in rats [12], no review has comprehensively mapped the use of control groups within preclinical research or assessed their impact on studies’ conclusions. Such a review would adequately equip scientists and clinicians with methods to plan or review preclinical experiments. Furthermore, it would highlight the challenges and limitations of using the different types of control groups in this context and identify potential opportunities for model improvement. Thus, this systematic review aims to summarize the different control groups used to compare new osteoregenerative test items investigated in preclinical femoral defect models in rats and to analyze potential pitfalls related to control groups to improve future preclinical research translation ability.

## 2. Materials and Methods

### 2.1. Search Strategy

The review protocol was registered in PROSPERO (Registration ID: CRD420261279773), consistently with the methodology applied throughout the review. A systematic literature review using a systematic review approach and following the “Preferred Reporting Items for Systematic Reviews and Meta-Analyses” (PRISMA) methodology was performed (Table S1—PRISMA Main Checklist, Table S2—PRISMA Abstract Checklist) [16]. The systematic search was constructed to identify preclinical studies using femoral defect models in rats to investigate new osteoregenerative test items. The search terms and additional methodology details are shown in Table S3—Search Strategy. The literature search was conducted through the health-related research database MEDLINE (PubMed) and the health and information technology database Embase for relevant literature.

### 2.2. Eligibility Criteria

Studies on in vivo investigation of new test items to improve bone healing in femoral defect models in rats were selected. To reduce selection bias, only studies evaluating the performance of a defined osteoregenerative test item in a living rat model were included. Studies that proposed solely surgical techniques for bone healing improvement without test items were excluded. All defect models created in the femur, regardless of the presence or absence of internal or external fixation, were eligible. Publications in peer-reviewed journals written in English between 1 January 2001 and 1 January 2023, were included. Narrative reviews, book chapters, conference abstracts, posters, and other non-peer-reviewed publications were excluded to ensure scientific rigor and reproducibility of reported findings. One reviewer (C.C.) independently reviewed all studies to be included.

### 2.3. Data Collection and Analysis

Data from all included studies was extracted into a standardized data extraction sheet (Table 1). Data from categories 9, 10, 14 and 15 were extracted by two reviewers (C.C., R.L.; Table 1). The remaining data was extracted by one reviewer (R.L). Dual-reviewer extraction was prioritized for categories directly linked to our main research questions (control groups, test items, and related outcomes) to minimize interpretation bias. For the remaining categories, single extraction was performed due to feasibility constraints (time and resources), but consistency was ensured through the use of a standardized data extraction sheet.

For the purposes of data analysis, rats younger than 20 weeks were classified as skeletally immature [17,18].

**Figure 1:**
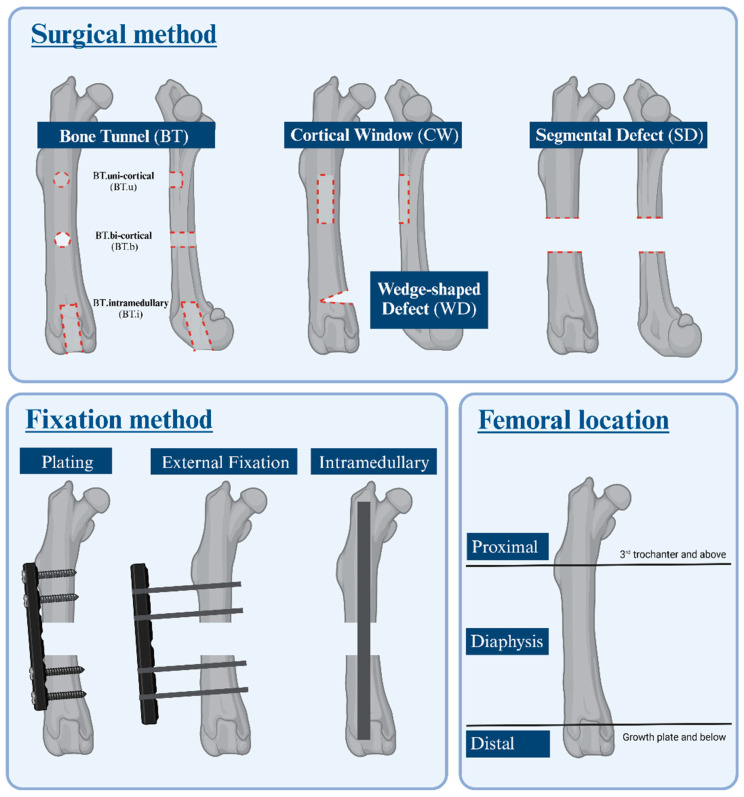
Schematic Representation of Surgical Methods, Fixation Methods, and Femoral Locations in Preclinical Rat Bone Defect Models.

The surgical method (bone tunnel (BT), cortical window (CW), wedge-shaped defect (WD), segmental defect (SD)), fixation method (plating, external fixation, intramedullary, no fixation), and femoral location (proximal, diaphysis, distal) of bone defects created in preclinical rat models were classified according to Sun et al. (Table 1, Figure 1) [19].

The number of rats reported refers to those that underwent an in vivo model. Rats used exclusively as tissue donors for in vitro investigations or bone grafts were not included in this count. Rats receiving the same treatment but sacrificed at different time points were considered as one group.

A negative control group was defined as an empty defect or treated with materials serving solely as an inactive carrier without impacting bone healing. A positive control group was defined as a group used for test item comparison using bone grafts or commercially proven bone substitutes used in the clinical treatment of large bone defects.

Bone graft origin could be the same individual (autologous bone graft, autograft), the same species (allogeneic bone graft, allograft), such as from rats of a similar or different strain, or a different species (xenogeneic bone graft, xenograft), such as from bovines or humans.

The test items’ outcomes were extracted from studies whenever a control group was included. These outcomes were based on the measured results and comparisons between the test item and the control groups. Critical findings related to inflammation or foreign body reactions associated with bone grafting were also extracted, whether they were part of a primary outcome, such as histological analysis, or arose from unexpected observation, such as exclusions of animals. In this review, the term “better healing” refers to superior outcomes as reported by the original study authors, which could include higher bone volume or density on imaging, improved histological scores, enhanced mechanical strength, or faster achievement of union. Because outcome measures, scoring systems, and time points varied substantially between studies, outcomes were not reclassified or standardized across studies. Instead, ‘better healing’ was recorded as relative superiority as reported by the original study authors within the context of their selected assessment method.

### 2.4. Study Quality Assessment and Risk of Bias

Reporting quality and risk of bias were evaluated using an integrated framework combining the SYRCLE Risk of Bias tool for animal intervention studies and the ARRIVE 2.0 “Essential 10” reporting items. ARRIVE items were used as structured indicators of methodological transparency and reporting completeness and were mapped to the corresponding SYRCLE bias domains to enable a qualitative assessment (Table 2).

Selection bias was assessed from the reporting of study design, control group definition and comparators, group allocation, and animal characteristics (strain, sex, age, weight, and comorbidities). Performance bias was evaluated based on the description of experimental procedures, observation period, and the overall completeness of procedural reporting. Detection bias was assessed from the clarity and objectivity of outcome measures, the reporting of statistical methods, and the description of blinding of surgery and/or outcome assessment. Attrition bias was evaluated based on the reporting of inclusion and exclusion criteria, complications or inflammatory reactions, and the handling of missing animals or data points. Each domain was graded qualitatively as low risk, some concerns, or high risk of bias, according to the completeness and consistency of reporting and the potential impact of methodological limitations on outcome interpretation.

### 2.5. Statistical Analysis

Data from all included studies were compiled into a standardized extraction sheet and analyzed descriptively in Microsoft Excel. A narrative review with descriptive statistics was used to summarize the wide range of preclinical studies on femoral defects in rats for bone healing improvements, as the diversity of models, controls, and outcomes made meta-analysis unsuitable.

To evaluate whether the type of control group influenced reported test item outcomes, we compared the proportion of studies reporting superior healing with the test item in studies using negative controls versus those using positive controls. A contingency table was constructed, and group comparison was performed using the Chi-square test for association. Odds ratios (OR) and 95% confidence intervals (CI) were calculated to quantify the strength of association. Statistical analyses were conducted in GraphPad Prism version 10.4.1 (GraphPad Software, San Diego, CA, USA), and *p*-values < 0.05 were considered statistically significant.

## 3. Results

### 3.1. Search and Screening

**Figure 2:**
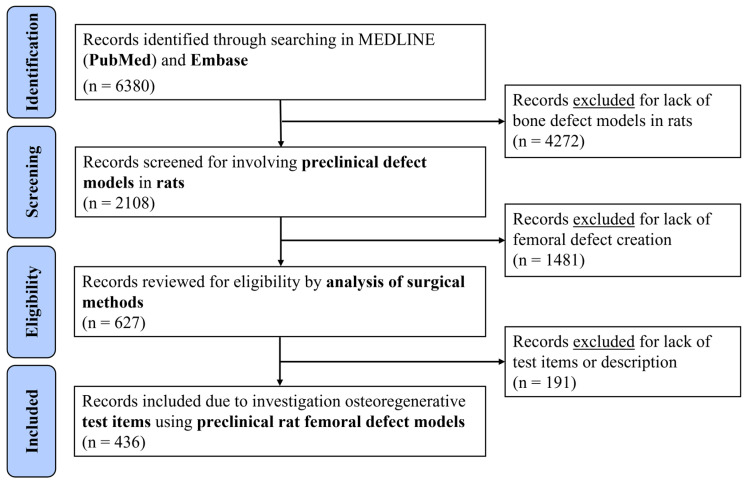
PRISMA Flowchart of the Systematic Review on Control Group Selection in Preclinical Rat Bone Defect Models. The PRISMA flowchart illustrates the systematic review process performed to investigate the control group selection in preclinical rat femoral defect models, summarizing the inclusion and exclusion criteria applied to identify relevant studies from January 2001 to January 2023.

The systematic search identified 6380 preclinical studies using femoral defect models in rats for the investigation of new osteoregenerative test items (Figure 2). After screening and eligibility assessment, 436 papers were included for the data extraction and analysis.

### 3.2. Publication Key Data

**Figure 3:**
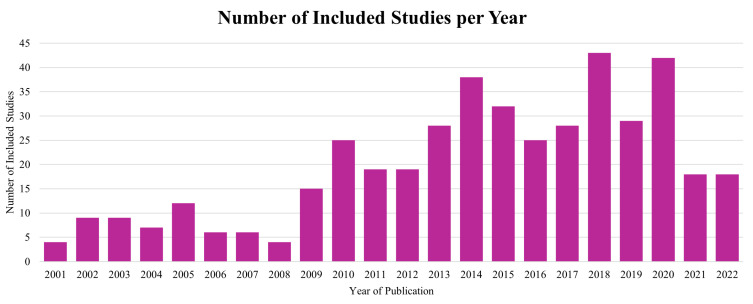
Annual Distribution of Included Studies in Preclinical Rat Bone Defect Models. Annual distribution of 436 studies on preclinical rat femoral defect models included in the review. Each bar represents the number of studies published per year, highlighting trends in research activity over time, with noticeable peaks around 2018 and 2020.

The number of relevant publications generally increased over the review period, with notable peaks observed in 2018 and 2020, which included 43 and 42 publications, respectively (Figure 3).

### 3.3. Demography of Rats Used

On average, 42 ± 22 rats and 4 ± 1 groups were used per study (Table 3), resulting in an average of 11 rats per group. Sprague-Dawley rats were used in approximately half of the studies (47%), followed by Wistar (28%) and Fischer rats (6%; Table 3).

**Figure 4:**
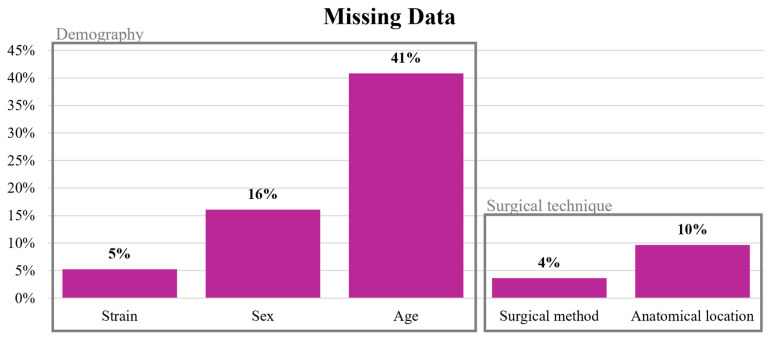
Incidence of Missing Data on Key Animal Model Characteristics in Included Studies. Bar chart showing the number of studies (out of 436) that did not report essential animal model characteristics: strain, sex, age, surgical method, and anatomical location. Percentages shown on top of each bar indicate the proportion of studies with missing data in each domain. Such omissions limit reproducibility, inter-study comparability, and the ability to conduct robust meta-analyses.

A large proportion of studies (45%, *n* = 198/436) used skeletally immature rats, while mature and aged rats were used in 14% of the studies (*n* = 61/436). Notably, the age of the rats was not reported in 41% of the studies (*n* = 178/436; Figure 4). The overall mean weight of the rats included was 331 g ± 191 g, with specific mean weights for Sprague-Dawley, Wistar and Fischer rats being 357 g ± 252 g, 307 g ± 101 g and 280 g ± 91 g, respectively. A total of 113 studies (26%) included comorbidities such as osteoporosis, immunosuppression, induced infection, or diabetes. Osteoporosis was the most commonly modeled comorbidity (Table 3), typically induced by ovariectomy. In infection models, Staphylococcus aureus was the sole pathogen used to induce local infection. Imaging outcomes encompassed diverse modalities, including micro-CT, radiographs, and other techniques, though reporting and methodology varied widely.

### 3.4. Surgical Technique (Surgical Method, Fixation Method, Femoral Location)

Segmental diaphyseal bone defects stabilized with plate and screws were the most commonly used surgical techniques (32%). Regarding specific surgical methods, segmental defects were the most frequently employed (43%), followed by uni-cortical bone tunnels (37%), bi-cortical bone tunnels (8%), cortical windows (7%), and wedge-shaped defects (1%). Femoral stabilization was not always required, with 55% of surgeries performed without stabilization, typically in cases like uni-cortical bone tunnels. When stabilization was performed, the use of bone plate and screws was the most common method (74%, *n* = 146/198), followed by external fixation (17%, *n* = 33/198) and intramedullary pin placement (9%, *n* = 17/198). The femoral diaphysis was the most frequent location for bone defects creation (71%), followed by defects located distal to the growth plate (17%). Only 2% of the studies created a defect in the 3rd trochanter and above. Notably, 10% of the included studies did not adequately describe the surgical location (Figure 4).

### 3.5. Control Groups

**Figure 5:**
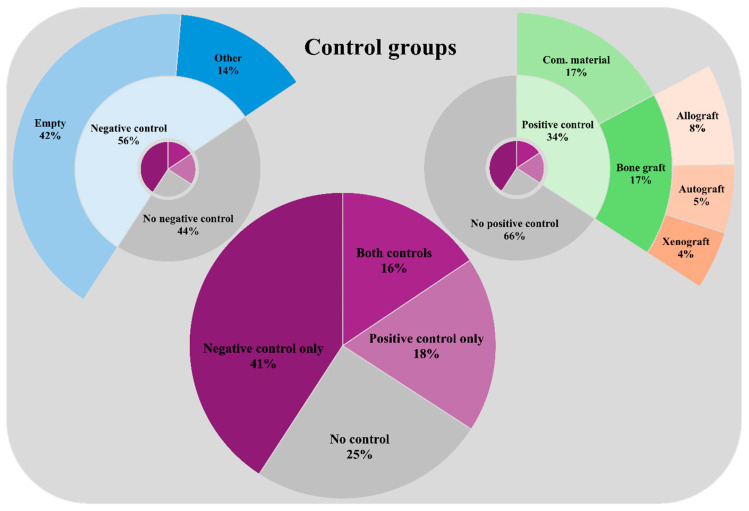
Control Groups Used in Preclinical Rat Bone Defect Studies. This figure presents a breakdown of control group strategies across the 436 included studies. The central pie chart categorizes studies by control group inclusion: negative controls only (*n* = 178), positive controls only (*n* = 82), both control types (*n* = 67), or no control group (*n* = 109). The top-left nested pie chart (blue) provides a detailed view of negative control types in applicable studies (*n* = 245), while the top-right nested pie chart (green and orange) shows the breakdown of positive control subtypes (*n* = 149), including types of bone grafts. Percentages reflect the proportion of studies within each subgroup.

The choice of control groups varied markedly across studies (Figure 5), with 25% (*n* = 109/436) of them not including any control. Of the studies that did include controls, 54% (*n* = 178/327) used only negative controls, 25% (*n* = 82/327) used only positive controls, and 20% (*n* = 67/327) included both. Consequently, 44% (*n* = 191/436) of the studies lacked negative control groups, and 66% (*n* = 287/436) lacked positive control groups. When negative controls were used, most frequently empty bone defects (75%, *n* = 184/245) or bone defects filled with inactive carriers (25%, *n* = 61/245) without influencing bone healing, such as titanium scaffolds, were used.

Positive controls typically involved bone defects filled with commercial bone substitutes (50%, *n* = 75/149) or bone grafts (50%, *n* = 74/149). Among studies using bone grafts as positive controls, allogeneic bone grafts were the most common (45%, *n* = 33/74), followed by autologous (30%, *n* = 22/74) and xenogeneic grafts (26%, *n* = 19/74).

### 3.6. Properties of Bone Graft

Overall, 31% (*n* = 135/436) of the included studies transplanted bone graft material and 17% (*n* = 74/436) of the included studies transplanted bone grafts as positive control.

**Figure 6:**
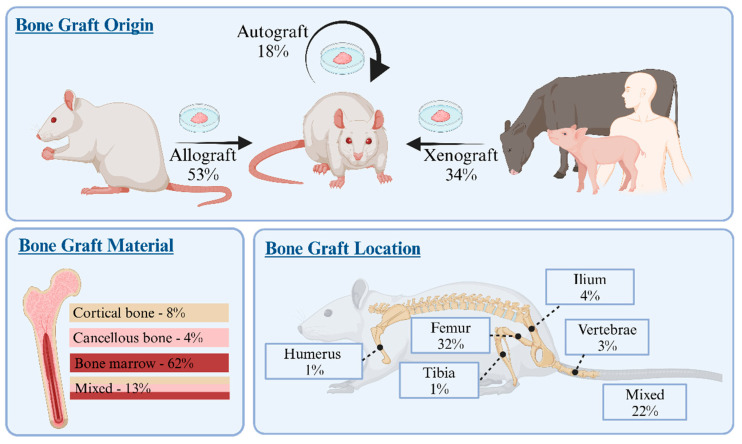
Properties of Bone Graft Used in Preclinical Rat Bone Defect Studies. Distribution of bone graft origins, materials, and harvesting sites in the 135 studies reporting graft use. The figure shows the frequency of each graft characteristic, with some studies including multiple graft types or sources. Percentages above bars reflect the proportion of studies reporting each attribute. Of note, 19% of studies did not report graft material and 39% did not report harvesting location, highlighting inconsistencies in reporting across the literature.

Allografts were the most commonly transplanted bone graft origin, used in 53% (*n* = 72/135) of the studies, with 45% (*n* = 33/74) employed as positive control (Figure 6). Most of the studies (82%, *n* = 59/72) used fresh grafts (sometimes combined with cell expansion techniques). Most of the studies (32%, *n* = 23/72) used a single donor rat for multiple recipients, with 72% (*n* = 52/72) using rats from the same rat strain as recipient and 11% (*n* = 8/72) using a different strain. Allogeneic bone grafts were most often harvested from the femur (39%, *n* = 28/72) or from multiple combined locations (38%, *n* = 27/72). Autografts were used least frequently as bone graft (18%, *n* = 24/135). As positive control, autografts were the second most frequently transplanted bone graft (30%, *n* = 22/74) (Figure 6). Autografts were typically harvested from the femur (67%, *n* = 16/24) and tail vertebrae (13%, *n* = 3/24), with crushed corticocancellous bone graft commonly used.

In the remaining studies, xenografts were used (34%, *n* = 46/135) (Figure 6), with grafts harvested from humans (65%, *n* = 30/46), bovines (17%, *n* = 7/46), or other species (20%, *n* = 9/46).

### 3.7. Comparison on Performance

**Figure 7:**
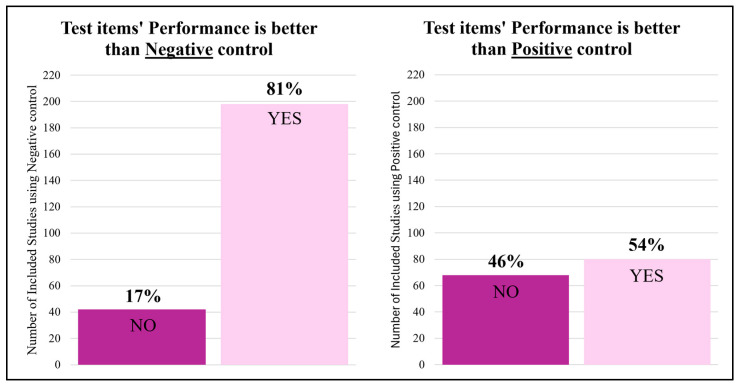
Influence of Control Group Type on Reported Test Item Outcomes. Comparison of healing outcomes for test items assessed against negative controls (left panel, *n* = 245) and positive controls (right panel, *n* = 149). Bars represent the number of studies reporting whether the test item outperformed the respective control group, with percentages indicating the proportion of studies within each category. “Better healing” refers to superior performance of the test item as reported by the original study authors, based on either qualitative or quantitative comparisons. Negative controls typically represent native healing (e.g., untreated defects), while positive controls include clinically validated treatments such as autografts, allografts, or commercial bone substitutes.

The reported performance of the test items appeared to be influenced by the choice of control group (Figure 7). In 81% of the studies that included control groups, the test items demonstrated better healing outcomes, defined as superior outcome measures as reported by the original study authors, compared to the negative control (*n* = 198/245), whereas 54% of the studies reported superior healing outcomes compared to positive controls (*n* = 80/149). Statistical analysis confirmed that test items were significantly more likely to demonstrate superior outcomes compared to negative controls than to positive controls (χ^2^ = 30.22, df = 1, *p* < 0.0001). Studies using negative controls were more likely to report test item superiority, with an odds ratio of 3.47 (95% CI: 2.22–5.38, *p* < 0.0001). Nevertheless, the assessment methods underlying these comparisons were heterogeneous, with studies relying on qualitative histological scoring, quantitative imaging parameters, biomechanical testing, or combinations thereof, which limits direct comparability across studies.

When comparing the test item to the bone graft used as a positive control, half of the studies (51%, *n*= 38/74) found the test item to perform better. More than half of the studies performed better compared to the commercial material used as positive control (56%, *n* = 42/75). A similar number of studies showed higher performance of the test item compared to autografts (59%, *n* = 13/22) and allografts (45%, *n* = 15/33).

### 3.8. Inflammation or Foreign Body Reaction Linked to Bone Grafting

Eighteen of the reviewed studies (13%, *n* = 18/135) reported inflammatory or foreign body reactions as side effects of bone grafting within bone defects, based on macro- or microscopic observations. No study reported such reactions due to autologous bone graft. Additionally, nearly one-third (30%, *n* = 40/135) of studies using bone grafts did not provide any information, either confirming or denying the presence of inflammatory or foreign body reactions.

### 3.9. Findings of the Study Quality Assessment and Risk of Bias Analysis

Across the included studies, most domains were judged as presenting moderate concern rather than clearly low or high risk of bias (Table 4). Domains directly associated with internal validity and bias control demonstrated substantial weaknesses. Randomization was not reported in most studies (67%, high concern), and blinding was almost universally absent (84%, high concern), indicating a high likelihood of selection and detection bias. Reporting of inclusion and exclusion criteria was frequently incomplete (74%, moderate concern), suggesting potential attrition-related bias that could not be fully evaluated. Information on animal characteristics showed mixed clarity (38%, lower concern; 46%, moderate concern), reflecting variability in baseline transparency and external validity. Although statistical methods were commonly included, the majority of studies were classified as moderate concern (76%) due to insufficient reporting of analytical assumptions or handling of data variability, suggesting risk of analytical and selective reporting bias.

## 4. Discussion

The growing number of publications on new biomaterials for enhancing or replacing bone healing underscores the ongoing challenge of treating large bone defects in orthopedic patients. This systematic review reveals significant variability in the choice of control groups in preclinical femoral defect models in rats, pointing to a lack of standardization. Such variation complicates cross-study comparisons and raises concerns about the validity of conclusions regarding the efficacy of osteoregenerative materials. Moreover, inconsistent use of bone grafts as positive controls further complicates outcome interpretation. These findings highlight the need to improve preclinical bone healing studies’ reliability and translational potential.

### 4.1. Animal Demographics

One critical aspect that influences the outcomes of preclinical orthopedic research is the selection of animal models, particularly in terms of strain, sex, and age. These demographic factors affect bone healing processes and have implications for the translational relevance of the findings for human clinical practice. The studies included in this review predominantly use Sprague-Dawley rats (47%), which may enhance inter-study comparability by reducing variability but could also introduce strain-specific biases that affect research outcomes. In addition to body size and weight differences [20], inter-strain phenotypic variations, such as bone geometry and fragility, are well-documented [21]. Despite this, the potential impact of strain selection on study outcomes in preclinical orthopedic research remains underexplored, highlighting the need for further investigation. Implementing multi-strain validation, as performed in other research fields [22], could help address this gap and improve the robustness of preclinical models.

Female rats were underrepresented in the included studies (25%) and only 1% of included studies reported using both sexes in their experiment. The investigation of sex differences in femoral defect healing is limited despite early preclinical studies demonstrating relevant differences in bone healing between sexes [23]. The significant discrepancy in sex representation is a possible limitation of studies. This imbalance could underestimate sex-related outcomes at the early stage of product development [24]. A conscious inclusion of both sexes would better model the target population of both female and male patients and could increase translational ability. This is particularly important given that women, especially those over 50, are at higher risk of fractures due to a greater prevalence of osteoporosis, underscoring the need for sex-specific considerations in bone healing research [25].

When selecting the animals, it is important to consider their age to align with the developmental stage of the target human population, as the translational ability from animals to humans improves when the model closely reflects clinical reality. Preclinical studies should use skeletally mature animals to model adult human bone healing accurately [26]. Despite this, skeletally mature and immature rats (14% and 45%) were used in the included studies, and 41% of studies failed to specify the age of the animals. Previous studies in rodents showed that age impacts bone integrity [17,27]. In addition, the healing capacities of bone differ between immature and mature individuals [28]. The definition of skeletal maturity in rats is not as straightforward as in humans, because the growth plate does not fully close in adult rats. Based on alternative assessments, rats could be classified as skeletally mature starting from 20 to 28 weeks of age, though studies have shown dynamic changes even beyond this period [27].

Weight variability (331 g ± 191 g) in included studies was influenced by multiple factors such as different strains, sex, and ages. Body weight plays a significant role in determining load stress on weight-bearing bones, which could impact bone healing outcomes. After surgery, rats are typically placed back into their cages with the ability to bear weight freely. Still, surgical techniques and fixation methods may have limitations in supporting animals with higher body weights. This further highlights the need to carefully consider rat demographics and related physiological factors, such as body weight, when designing studies.

### 4.2. Femoral Location and Surgical Techniques

All femoral locations, including the diaphysis (71%), proximal metaphysis and epiphysis (2%), and distal metaphysis and epiphysis (17%), were used to create bone defects in the included studies. The femoral location selected depends, among other factors, on the surgical method and accessibility. Segmental defects are typically placed in the diaphysis to allow for proper fixation, while bone tunnels can be created in other regions of the bone. Nevertheless, bone structure and regeneration capacity vary across different bone regions [29,30]. The cancellous bone is located primarily in the metaphyses of long bones and provides the best regenerative capacity [7]. In contrast, the diaphysis consists largely of cortical bone with bone marrow-filled tunnels [31]. Since long bone fractures in adult patients occur most frequently in the metaphysis, metaphyseal defect models are highly relevant in preclinical orthopedic research [26,32]. Nonetheless, the metaphysis and epiphysis were not frequently used locations in the included studies (19%).

A segmental diaphyseal femoral defect fixed using a plate and screws was the preferred surgical technique in the reviewed studies (32%). Nevertheless, this review highlights significant variability in the surgical techniques used to investigate the healing properties of new biomaterials, even though many studies appeared to follow previously established study designs [12]. The difficulty of the surgical methods varied between studies and reflects different required levels of surgical expertise [12]. Furthermore, the surgical protocols published were often insufficiently detailed to assess how reproducible the techniques were. Ensuring reproducible bone defect size is the first aspect of a standardized defect. In that line of thinking, surgical techniques using instruments and implants that enhance reproducibility are crucial and should be prioritized. For example, using jigs for osteotomy or drilling helps create uniform defects and reduces intra- and inter-study variability. The size of the defect, whether in terms of diameter, length, or both, was another significant source of variation between studies. Compared to smaller defects known to heal naturally, critical size defects mirror the clinical challenges of large bone defects and enhance the relevance of findings for clinical translation. The implant systems, such as internal (plating, nailing), external fixation, and materials like titanium and PEEK, are similar to those used clinically in humans, enhancing translational potential when used [33,34,35]. The implant-provided stability influences the defect healing [36], with most fixation methods offering varying degrees of relative stability [37], much like those used in human multi-fragmentary fractures [38], Standardization of fixation and defect stability is crucial for inter-study comparability. To this aim, following established, concept-proved defect models and bone fixation is essential to create more standardized and comparable data [39]. New pilot studies developing novel defect models should be precisely reported to ensure reproducibility and allow for improvement if needed [19].

### 4.3. Impact of Control Group and Bone Graft Selection

The findings from this systematic review highlight the influence of control group selection on the perceived performance and efficacy of new test items on bone healing. The choice of control groups dictates the performance level the test item must reach, with test items from included studies often appearing superior to negative controls but potentially underperforming when compared to positive controls. This can be partially attributed to the role of negative controls, representing native healing, which is easier to surpass than the current clinical standard. If a test item performs worse than the positive control, a negative control can verify whether the test item is supportive in any way. The positive control serves as a benchmark for evaluating the degree of the supportive effects of the test item. However, suppose the benchmark is set too low due to an inadequate choice of control group, in that case, it may lead to statistically significant differences that give the false impression of superior test item performance. Using no control groups makes performance analysis of the test item impossible or unreliable. Leaving out a positive control group avoids the risk of underperforming, but such comparisons are essential to determine whether the test item is worth further investigation or clinical trials. In our dataset, test items more frequently outperformed negative controls than positive controls. This observation was supported by formal statistical analysis: test items were 3.47 times more likely to show superior outcomes when compared to negative controls (95% CI: 2.22–5.38), highlighting a potential bias in perceived efficacy when only negative controls are used. These results emphasize that control group selection is not a neutral choice but a determinant of the apparent success of an intervention. Future preclinical study designs should explicitly justify their choice of control and adopt consistent definitions across studies to minimize interpretation bias. Establishing standardized protocols for when and how to use negative versus positive controls would enhance methodological transparency, increase the reliability of comparisons across studies, and guide evidence-based progression toward clinical translation.

The gold standard in human clinics is the autograft, and cancellous autografts could be considered the highest benchmarks, a test item has to meet. In human surgery, cancellous bone is preferred for its high osteoconductive, osteoinductive, and osteogenic properties, though it lacks structural support [7]. This issue can be solved by using a scaffold or corticocancellous graft [7]. Cortical grafts provide stability but lack regenerative capacity [7]. The iliac crest remains the gold standard for harvesting cancellous or corticocancellous grafts in humans [7,40,41,42]. Still, it is rarely used in rat surgeries, as highlighted in this review (4%). Techniques like the reaming–irrigator–aspirator (RIA) system are also standard in humans [41].

Control groups should also be selected to ensure standardization and objectivity. The lack of standardization, especially for positive controls, increases the risk of misinterpretation of new test items’ performances. As shown in this review, there is a high degree of heterogeneity in the selection of control groups, especially positive controls. The heterogeneity of positive controls used in preclinical bone studies in rats contrasts with the single gold standard in human medicine, the autologous cancellous bone graft [3,4]. In addition to the variations between bone graft types and origins and commercial bone substitutes, the differences in the processing of bone grafts further contribute to this variability. Our findings support the need for greater consensus and harmonization in the selection of positive controls in preclinical models. Standardized criteria for control group composition, aligned with clinical benchmarks, would reduce heterogeneity, support robust statistical comparisons, and improve reproducibility and interpretability of findings.

Autografts are rarely used in rat models, possibly due to the complexity of standardized harvesting techniques, which have only recently been described [43,44]. The small size of rats might limit the feasibility of harvesting autografts with human-like qualities. Further research is needed to find possible surgical techniques to produce autograft quality similar to that of a human autograft [44]. Another reason for avoiding autografts could be expected side effects, such as pain from the harvest site, which might affect weight-bearing and bone healing [45]. The intensity of side effects varies depending on the harvest site and species [45,46]. For instance, sheep and rabbits tolerate iliac crest harvesting quite well, while humans usually report more pain after the procedure [47,48,49,50]. Limited data regarding postoperative pain after bone graft harvesting is available in rats and warrants further investigation.

Other graft types, such as allografts and xenografts, replace autografts in some preclinical studies. Nevertheless, they could induce inflammatory and foreign body reactions [50], which is consistent with reported inflammatory and foreign body reactions after bone grafting in some of the included studies (*n* = 18/135). On the contrary, negative tissue responses were not reported for autologous tissue in the included studies. Using immunocompromised rats to prevent foreign body reaction, as reported in 42% (*n* = 30/72) of studies using allografts, reduces the comparability to other studies with non-compromised rats and the possibility of the translation ability of the results to clinical practice. Instead of using immunocompromised animal models to mitigate the adverse side effects of allografts, developing feasible techniques for standardized, high-quality autografts would better replicate human clinical conditions.

### 4.4. Assessment Methods of Healing

The assessment of bone healing across the included studies was highly heterogeneous. Histology was the most frequently reported outcome (89%), followed by imaging (29%) and mechanical testing (27%). Imaging encompassed various modalities, including micro-CT, radiographs, and other techniques, but reporting and methodology differed markedly between studies. Likewise, mechanical testing methods varied, with studies applying three-point bending, torsion, or compression at different time points. Importantly, the definition of “healing” was inconsistent: some authors reported qualitative histological improvements, others quantified bone volume or density using imaging, while a minority measured biomechanical strength. This variability complicates direct comparisons and highlights the absence of a standardized definition of healing or union in preclinical rat femoral defect models. Developing consensus criteria for outcome assessment would greatly improve comparability and translational value in future research.

Importantly, the definition of ‘better healing’ in this review reflects the conclusions drawn by the original study authors and is therefore influenced by the outcome measures selected and their interpretation. Studies using qualitative histological assessments may report improvement differently than those relying on quantitative imaging or biomechanical endpoints. This subjectivity, combined with inconsistent reporting and lack of standardized success criteria, likely contributes to the high proportion of studies reporting positive effects, particularly when negative controls are used. These findings highlight the need for consensus on primary outcome definitions and minimal reporting standards in preclinical bone defect models.

### 4.5. Risk of Bias

Although procedural aspects of the animal models were generally well documented, the quality assessment indicates that important safeguards to minimize bias were often insufficiently reported or not implemented. In particular, limited reporting of randomization and blinding, together with incomplete documentation of exclusions, raises concern that selection, detection, and attrition bias may have influenced outcomes in a substantial proportion of studies. These methodological gaps reduce confidence in the internal validity of reported effects and may partly explain variability and challenges in reproducing findings across preclinical bone healing studies. Rather than reflecting isolated reporting omissions, the pattern observed across domains suggests that bias-control practices remain inconsistently embedded in experimental design within this field. Strengthening routine use and transparent reporting of randomization, blinding, and exclusion handling in addition to detailed procedural reporting should therefore be considered a priority to improve the interpretability and translational relevance of preclinical bone defect models.

### 4.6. Limitations

This review has several methodological limitations that should be acknowledged. First, the initial title and abstract screening was conducted primarily by a single reviewer, which may have introduced selection bias despite subsequent full-text verification and cross-checking of uncertain cases. A dual-reviewer screening process would have strengthened reliability and minimized the risk of study exclusion errors. Second, a single-extraction process was used for certain data fields, which may have introduced bias in data interpretation. The insufficient reporting of essential information regarding the animal model in the included studies led to potential biases from unreported data. Additionally, the reliance on author-defined outcome superiority (‘better healing’) introduces subjectivity that could not be resolved due to heterogeneity in outcome measures, scoring systems, and follow-up durations across studies.

Although a qualitative risk-of-bias and reporting assessment was performed using an integrated ARRIVE–SYRCLE framework, this approach is inherently dependent on what was reported by the original authors and therefore reflects reporting quality as a proxy for true methodological rigor. In several domains, missing information had to be classified as “high/unclear concern,” which may overestimate or underestimate the actual risk of bias. The qualitative nature of the assessment also required domain-level judgment rather than quantitative scoring or weighting across studies. Heterogeneity in surgical techniques, graft processing, control group selection, and outcome definitions limited the comparability of studies and precluded meta-analytic synthesis. Finally, the PROSPERO registration was completed retrospectively after the systematic review was already in progress; however, the registered protocol reflects the procedures that were followed during the review.

### 4.7. Recommendations for Future Research

Future research could be enhanced by consistently reporting key demographic data points, such as strain, sex, age, and weight of the rats used [26]. These biological parameters can influence bone mechanical properties, and, consequently, defect healing. Cross-comparison between studies is limited when its basic information is missing. Studies should prioritize the reporting of standardized and comprehensive data to allow them to serve as valuable knowledge points or be included in broader meta-analyses. Bigender and multi-strain studies using skeletal mature rats appear purposeful, although financial and time-related constraints must be considered. The 3R principles (Reduction, Replacement, Refinement) should also be considered and followed when planning further experiments. Therefore, selecting rat demographics carefully is essential to using as many rats as necessary but as few as possible to produce reliable data. Further investigation into surgical techniques for standardized cancellous autologous bone graft harvesting is needed to maintain alignment with the gold standard in humans for use as a positive control in rats. The inclusion of valid control groups is essential in planning future preclinical studies. Collaborative efforts to create shared guidance on control group selection in bone healing studies could be highly beneficial to the field. At a minimum, preclinical rat femoral defect studies evaluating new osteoregenerative test items should include both a negative control (e.g., an empty defect or inert carrier) to document native healing and a positive control aligned with clinical standards, ideally a cancellous autologous bone graft or a well-justified surrogate when autografting is not feasible. Clear justification for control group selection and transparent reporting of control characteristics should be considered essential requirements for study design and peer review. These recommendations would significantly improve inter-study comparability and enhance the translational ability to clinical use. In addition, the risk-of-bias assessment from this review highlights the need for future studies to systematically implement and transparently report randomization, blinding of outcome assessment, and handling of exclusions, as these measures remain critical to strengthening internal validity and reducing the likelihood of selection, detection, and attrition bias.

## 5. Conclusions

In conclusion, the selection of control groups in preclinical bone defect models impacts the reported performance of new biomaterials. Our review shows that test items frequently appear more effective when compared to negative controls than to positive controls, highlighting the importance of careful group selection. This variability emphasizes the need for standardized protocols to improve preclinical research’s reliability and translational ability. Future studies should prioritize optimizing control group selection to reflect clinical realities better, ultimately aiding in developing effective and commercially viable bone healing biomaterials.

## Figures and Tables

**Table 1:** Extracted data from the studies included in the review.

Category		Description
**(1)**	**Publication key data**	(a) Primary author
		(b) Year published
		(c) Journal published
		(d) Title
**(2)**	**Demography of rats used**	(a) Strain
		(b) Sex
		(c) Average age (weeks)
		(d) Average weight (grams)
**(3)**	**Surgical method**	(a) Segmental defect
		(b) Bone tunnel (Uni-cortical, Bi-cortical, Intramedullary)
		(c) Cortical window
		(d) Wedge-shaped defect
**(4)**	**Fixation method**	(a) Plating
		(b) External fixation
		(c) Intramedullary
		(d) No fixation
**(5)**	**Femoral location**	(a) Proximal (3rd trochanter and above)
		(b) Diaphysis (Below 3rd trochanter to the distal growth plate)
		(c) Distal (Growth plate and below)
**(6)**	**Model comorbidities**	(a) Osteoporosis
		(b) Immunocompromised
		(c) Induced infection
		(d) Diabetes
**(7)**	**Total number of rats used**	
**(8)**	**Total number of groups used**	
**(9)**	**Control group(s)**	(a) Negative control group
		(b) Positive control group
**(10)**	**Test item investigated**	
**(11)**	**Properties of bone graft, when applicable**	(a) Origin (Autograft, Allograft, Xenograft)
		(b) Material (Bone marrow, Cancellous bone, Cortical bone, Mixed, Other)
		(c) Location origin (Humerus, Ilium, Femur, Tibia, Vertebrae, Mixed, Other)
		(d) Processing (Fresh, Frozen, Demineralized, Other)
		(e) Number of donors per recipient
		(f) Demography of donor (Strain, Average age, Average weight)
**(12)**	**Observation period (weeks)**	
**(13)**	**Outcomes evaluated**	(a) Histology
		(b) Imaging (X-ray, MicroCT, Other)
		(c) Mechanical testing
		(d) Functional testing
		(e) Other
**(14)**	**Outcomes comparing test items with the study’s controls**	
**(15)**	**Critical findings regarding inflammation or foreign body reaction linked to bone grafting**	

**Table 2:** Integrated ARRIVE 2.0–SYRCLE qualitative risk-of-bias assessment framework for included preclinical rat femoral defect studies.

ARRIVE Essential 10 Item	Indicator	Qualitative Judgment	Mapped SYRCLE Domain
**1. Study design**	Control group clearly defined & justified	**Lower concern**	Selection bias (group comparability)
	Control present but weakly defined or only positive/negative control without justification	**Moderate concern**	
	No control/unclear comparator	**High concern**	
**2. Sample size (reporting transparency)**	Group & total *N* clearly reported	**Lower concern**	Reporting bias
	Numbers partially reported (groups or total only)	**Moderate concern**	
	*N* not clearly reported	**High concern**	
**3. Inclusion & exclusion criteria**	Exclusions/complications reported transparently and complete reporting of data points used in analysis	**Lower concern**	Attrition bias
	Indications of exclusions but unclear handling or partial reporting of exact *n* reported per group	**Moderate concern**	
	No mention of attrition/exclusions	**High concern**	
**4. Randomization**	Randomization explicitly stated (method described or clearly indicated)	**Lower concern**	Selection bias (sequence generation)
	Unclear or not reported	**High concern**	
**5. Blinding**	Blinding stated and fully blinded (surgery and analysis)	**Lower concern**	Detection bias
	Blinding stated and partially blinded (surgery or analysis)	**Moderate concern**	
	Not reported or explicitly not blinded	**High concern**	
**6. Outcome measures**	Outcomes clearly defined and quantitative	**Lower concern**	Detection bias (selective reporting)
	Mix of descriptive and quantitative outcomes	**Moderate concern**	
	Outcomes poorly defined	**High concern**	
**7. Statistical methods**	Statistical methods explicitly described in Methods section	**Lower concern**	Detection bias
	Some statistics reported but unclear handling or missing assumption checks/handling of violations	**Moderate concern**	
	No statistical methods reported or no significance values despite interpretive claims	**High concern**	
**8. Experimental animals**	Full demographics reported (strain, sex, age, weight, comorbidities, donor demographics where relevant)	**Lower concern**	External validity/Other bias
	Missing one demographic field	**Moderate concern**	
	Minimal demographic reporting	**High concern**	
**9. Experimental procedures (surgery and/or graft)**	Model & procedure clearly described (surgical method class, fixation method, femur location, observation period, graft origin & processing when present)	**Lower concern**	Performance bias
	Missing one or two procedural details	**Moderate concern**	
	Procedure insufficiently described	**High concern**	

**Table 3:** Demographics of Rats and Study Group Outcomes in Included Studies. Data are presented as percentages (%), absolute numbers (*n*/N), or means ± standard deviations where applicable.

Demographics of Rats and Study Group Outcomes		Data	
**Strain**	Sprague-Dawley (SD)	47%	*n* = 207/436
	Wistar (Wi)	28%	*n* = 123/436
	Fischer (F344)	6%	*n* = 27/436
	Other	14%	*n* = 59/436
**Sex**	Male only	58%	*n* = 251/436
	Female only	25%	*n* = 110/436
	Both	1%	*n* = 5/436
**Average age (weeks)**		16 ± 13	
**Average weight (grams)**		331 ± 191	
**Model comorbidities**	Osteoporosis	14%	*n* = 59/436
	Immunocompromised	8%	*n* = 36/436
	Induced infection	3%	*n* = 14/436
	Diabetes	1%	*n* = 4/436
**Total number of rats used (#)**		42 ± 22	
**Total number of groups used (#)**		4 ± 1	
**Outcomes evaluated**	Histology	89%	*n* = 390/436
	Imaging	29%	*n* = 128/436
	Mechanical testing	27%	*n* = 117/436
	Functional testing	<1%	*n* = 2/436

**Table 4:** Distribution of qualitative risk-of-bias judgements across ARRIVE–SYRCLE assessment domains (% of included studies).

Domain	Lower Concern	Moderate Concern	High Concern
**Study design**	17%	**58%**	25%
**Sample size (reporting transparency)**	**76%**	10%	14%
**Inclusion & exclusion criteria**	12%	**74%**	14%
**Randomization**	33%		**67%**
**Blinding**	1%	15%	**84%**
**Outcome measures**	**59%**	36%	5%
**Statistical methods**	12%	**76%**	11%
**Experimental animals**	38%	**46%**	16%
**Experimental procedures**	**70%**	19%	11%

## Data Availability

The datasets generated during and/or analyzed during the current study are available from the corresponding author on reasonable request.

## Supplementary Materials

The following supporting information can be downloaded at: https://www.mdpi.com/article/10.3390/jfb17020066/s1. Table S1—PRISMA Main Checklist; Table S2—PRISMA Abstract Checklist; Table S3—Search Strategy.

## Abbreviations

The following abbreviations are used in this manuscript:

PRISMA	Preferred Reporting Items for Systematic Reviews and Meta-Analyses
BT	Bone tunnel
CW	Cortical window
WD	Wedge-shaped defect
SD	Segmental defect
OR	Odds ratios
CI	Confidence intervals
RIA	Reaming–irrigator–aspirator
