# Supplementary material for: Control Group Selection in Preclinical Rat Bone Defect Models: A Systematic Review
Source: J Funct Biomater. 2026 Jan 28;17(2):66. doi: 10.3390/jfb17020066 (PMC12942410; doi:10.3390/jfb17020066)
Supplement: Supplementary file 1 [file jfb-17-00066-s001.zip › jfb-4060927-supplementary.pdf]

Control Group Selection in Preclinical Rat Bone Defect Models: A Systematic Review

Lotta Reimann, Emma Marchionatti, Adrian Steiner, Stephan Zeiter and Caroline Constant

Table S1 - PRISMA Main Checklist

Topic	No.	Item	Location where item is reported
**TITLE**			
**Title**	1	Identify the report as a systematic review.	Control Group Selection in Preclinical Rat Bone Defect Models: A Systematic Review
**ABSTRACT**			
**Abstract**	2	See the PRISMA 2020 for Abstracts checklist	Annex 2 - PRISMA Abstract Checklist
**INTRODUCTION**			
**Rationale**	3	Describe the rationale for the review in the context of existing knowledge.	A review of preclinical bone defect models in rats and current research practices regarding control group selection for investigation of new biomaterials is essential, given the need to standardize research protocols and improve the translation ability of such studies to commercial use in humans.
**Objectives**	4	Provide an explicit statement of the objective(s) or question(s) the review addresses.	This systematic review aims to summarize the different control groups used to compare new osteoregenerative test items investigated in preclinical femoral defect models in rats and to analyze potential pitfalls related to control groups to improve future preclinical research translation ability.
**METHODS**			
**Eligibility criteria**	5	Specify the inclusion and exclusion criteria for the review and how studies were grouped for the syntheses.	Studies on *in vivo* investigation of new test items to improve bone healing in femoral defect models in rats were selected. Studies that proposed solely surgical techniques for bone healing improvement without test items were excluded. All femoral defect models with or without internal or external fixation were included. Publications in peer-reviewed journals written in English between January 1st, 2001, and January 1st, 2023, were included. Non-peer-reviewed references such as journal reviews, book chapters, conference abstracts, and posters were excluded. One reviewer (CC) independently reviewed all studies to be included.
**Information sources**	6	Specify all databases, registers, websites, organisations, reference lists and other sources searched or consulted to identify studies. Specify the date when each source was last searched or consulted.	The systematic search was constructed to identify preclinical studies using femoral defect models in rats to investigate new osteoregenerative test items. The search terms and additional methodology details are shown in Annex 3 - Search Strategy. The literature search was conducted through the health-related research database MEDLINE (PubMed) and the health and information technology database Embase for relevant literature.

Topic	No.	Item	Location where item is reported
**Search strategy**	7	Present the full search strategies for all databases, registers and websites, including any filters and limits used.	Annex 3 – Search Strategy
**Selection process**	8	Specify the methods used to decide whether a study met the inclusion criteria of the review, including how many reviewers screened each record and each report retrieved, whether they worked independently, and if applicable, details of automation tools used in the process.	One reviewer (CC) independently reviewed all studies to be included.
**Data collection process**	9	Specify the methods used to collect data from reports, including how many reviewers collected data from each report, whether they worked independently, any processes for obtaining or confirming data from study investigators, and if applicable, details of automation tools used in the process.	Data from all included studies was extracted into a standardized data extraction sheet. The control group(s), the test item investigated, outcomes comparing test items with the study's controls, and critical findings regarding inflammation or foreign body reaction linked to bone grafting were extracted independently by two reviews (CC, RL; Table 1). The remaining data related to the study methodology was extracted by one reviewer (RL).

**Data items**	10a	List and define all outcomes for which data were sought. Specify whether all results that were compatible with each outcome domain in each study were sought (e.g. for all measures, time points, analyses), and if not, the methods used to decide which results to collect.	Data from all included studies was extracted into a standardized data extraction sheet.<br>(1) Publication key data<br>a) Primary author<br>b) Year published<br>c) Journal published<br>d) Title<br>(2) Demography of rats used<br>a) Strain<br>b) Sex<br>c) Average age (weeks)<br>d) Average weight (grams)<br>(3) Surgical method<br>a) Segmental defect<br>b) Bone tunnel (Uni-cortical, Bi-cortical, Intramedullary)<br>c) Cortical window<br>d) Wedge-shaped defect<br>(4) Fixation method<br>a) Plating<br>b) External fixation<br>c) Intramedullary<br>d) No fixation<br>(5) Femoral location<br>a) Proximal (3rd trochanter and above)<br>b) Diaphysis (Below 3rd trochanter to distal growth plate)<br>c) Distal (Growth plate and below)<br>(6) Model comorbidities<br>a) Osteoporosis<br>b) Immunocompromised<br>c) Induced infection<br>d) Diabetes<br>(7) Total number of rats used<br>(8) Total number of groups used<br>(9) Control group(s)<br>a) Negative control group<br>b) Positive control group<br>(10) Test item investigated<br>(11) Properties of bone graft, when applicable<br>a) Origine (Autograft, Allograft, Xenograft)<br>b) Material (Bone marrow, Cancellous bone, Cortical bone, Mixed, Other)<br>c) Location origin (Humerus, Ilium, Femur, Tibia, Vertebrae, Mixed, Other)<br>d) Processing (Fresh, Frozen, Demineralized, Other)<br>e) Number of donors per recipient<br>f) Demography of donor (Strain, Average age, Average weight)<br>(12) Observation period (weeks)<br>(13) Outcome evaluated<br>a) Histology<br>b) Imaging (X-ray, MicroCT, Other)<br>c) Mechanical testing<br>d) Functional testing<br>e) Other<br>(14) Outcomes comparing test items with the study's controls<br>(15) Critical findings regarding inflammation or foreign body reaction linked to bone grafting

Topic	No.	Item	Location where item is reported
	10b	List and define all other variables for which data were sought (e.g. participant and intervention characteristics, funding sources). Describe any assumptions made about any missing or unclear information.	Table 1
**Study risk of bias assessment**	11	Specify the methods used to assess risk of bias in the included studies, including details of the tool(s) used, how many reviewers assessed each study and whether they worked independently, and if applicable, details of automation tools used in the process.	Risk of bias and reporting quality were assessed using a qualitative framework integrating SYRCLE's risk-of-bias domains with the ARRIVE 2.0 Essential 10 reporting items. ARRIVE indicators were mapped to corresponding SYRCLE domains (selection, performance, detection, attrition, and other bias). Each study was evaluated at the domain level and classified as low risk, some concerns, or high/unclear risk of bias based on reporting completeness and methodological transparency. The assessment was performed by one reviewer using predefined criteria, and results were summarized descriptively across studies.
**Effect measures**	12	Specify for each outcome the effect measure(s) (e.g. risk ratio, mean difference) used in the synthesis or presentation of results.	NA
**Synthesis methods**	13a	Describe the processes used to decide which studies were eligible for each synthesis (e.g. tabulating the study intervention characteristics and comparing against the planned groups for each synthesis (item 5)).	Quantitative assessment of the data items extracted.
	13b	Describe any methods required to prepare the data for presentation or synthesis, such as handling of missing summary statistics, or data conversions.	Data in the different units were converted (e.g. 4 months of age = 16 weeks of age).
	13c	Describe any methods used to tabulate or visually display results of individual studies and syntheses.	Using Microsoft Excel and BioRender.

Topic	No.	Item	Location where item is reported
	13d	Describe any methods used to synthesize results and provide a rationale for the choice(s). If meta-analysis was performed, describe the model(s), method(s) to identify the presence and extent of statistical heterogeneity, and software package(s) used.	Not performed.
	13e	Describe any methods used to explore possible causes of heterogeneity among study results (e.g. subgroup analysis, meta-regression).	A subgroup analysis of the four most frequent surgical models used to compare the control groups.<br>A subgroup analysis of the three most frequent bone graft origins used to evaluate their impact on the study results defined by the comparison of test items with control groups and potential pitfalls related to control groups.
	13f	Describe any sensitivity analyses conducted to assess robustness of the synthesized results.	Not performed.
**Reporting bias assessment**	14	Describe any methods used to assess risk of bias due to missing results in a synthesis (arising from reporting biases).	Not performed.
**Certainty assessment**	15	Describe any methods used to assess certainty (or confidence) in the body of evidence for an outcome.	Not performed.
**RESULTS**			
**Study selection**	16a	Describe the results of the search and selection process, from the number of records identified in the search to the number of studies included in the review, ideally using a flow diagram.	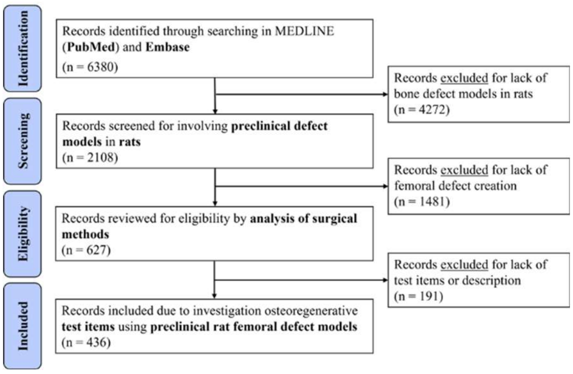<br>*Figure 2: PRISMA Flowchart of the Systematic Review on Control Group Selection in Preclinical Rat Bone Defect Models*
	16b	Cite studies that might appear to meet the inclusion criteria, but which were excluded, and explain why they were excluded.	NA

Topic	No.	Item	Location where item is reported
**Study characteristics**	17	Cite each included study and present its characteristics.	Table 3
**Risk of bias in studies**	18	Present assessments of risk of bias for each included study.	Table 4
**Results of individual studies**	19	For all outcomes, present, for each study: (a) summary statistics for each group (where appropriate) and (b) an effect estimate and its precision (e.g. confidence/credible interval), ideally using structured tables or plots.	NA
**Results of syntheses**	20a	For each synthesis, briefly summarise the characteristics and risk of bias among contributing studies.	NA
	20b	Present results of all statistical syntheses conducted. If meta-analysis was done, present for each the summary estimate and its precision (e.g. confidence/credible interval) and measures of statistical heterogeneity. If comparing groups, describe the direction of the effect.	Not performed.
	20c	Present results of all investigations of possible causes of heterogeneity among study results.	NA
	20d	Present results of all sensitivity analyses conducted to assess the robustness of the synthesized results.	NA
**Reporting biases**	21	Present assessments of risk of bias due to missing results (arising from reporting biases) for each synthesis assessed.	Missing results causes a risk of bias arising from reporting bias. *"Figure 4: Incidence of Missing Data on Key Animal Model Characteristics in Included Studies*" show a few examples for unreported data.
**Certainty of evidence**	22	Present assessments of certainty (or confidence) in the body of evidence for each outcome assessed.	Not performed.
**DISCUSSION**			

Topic	No.	Item	Location where item is reported
**Discussion**	23a	Provide a general interpretation of the results in the context of other evidence.	Yes
	23b	Discuss any limitations of the evidence included in the review.	Yes
	23c	Discuss any limitations of the review processes used.	Yes
	23d	Discuss implications of the results for practice, policy, and future research.	Yes
**OTHER INFORMATION**			
**Registration and protocol**	24a	Provide registration information for the review, including register name and registration number, or state that the review was not registered.	Not performed.
	24b	Indicate where the review protocol can be accessed, or state that a protocol was not prepared.	Not performed
	24c	Describe and explain any amendments to information provided at registration or in the protocol.	Not performed.
**Support**	25	Describe sources of financial or non-financial support for the review, and the role of the funders or sponsors in the review.	There are no external funders or sponsors involved.
**Competing interests**	26	Declare any competing interests of review authors.	There are no competing interests or disclosures to be declared by any of the authors.
**Availability of data, code and other materials**	27	Report which of the following are publicly available and where they can be found: template data collection forms; data extracted from included studies; data used for all analyses; analytic code; any other materials used in the review.	NA

Table S2 - PRISMA Abstract Checklist

Topic	No.	Item	Reported?
**TITLE**			
**Title**	1	Identify the report as a systematic review.	Yes
**BACKGROUND**			
**Objectives**	2	Provide an explicit statement of the main objective(s) or question(s) the review addresses.	Yes
**METHODS**			
**Eligibility criteria**	3	Specify the inclusion and exclusion criteria for the review.	Yes
**Information sources**	4	Specify the information sources (e.g. databases, registers) used to identify studies and the date when each was last searched.	Yes
**Risk of bias**	5	Specify the methods used to assess risk of bias in the included studies.	Yes
**Synthesis of results**	6	Specify the methods used to present and synthesize results.	Yes
**RESULTS**			
**Included studies**	7	Give the total number of included studies and participants and summarise relevant characteristics of studies.	Yes
**Synthesis of results**	8	Present results for main outcomes, preferably indicating the number of included studies and participants for each. If meta-analysis was done, report the summary estimate and confidence/credible interval. If comparing groups, indicate the direction of the effect (i.e. which group is favoured).	Yes
**DISCUSSION**			
**Limitations of evidence**	9	Provide a brief summary of the limitations of the evidence included in the review (e.g. study risk of bias, inconsistency and imprecision).	Yes
**Interpretation**	10	Provide a general interpretation of the results and important implications.	Yes
**OTHER**			
**Funding**	11	Specify the primary source of funding for the review.	Yes
**Registration**	12	Provide the register name and registration number.	Yes

Table S3 - Search Strategy

The search terms were selected to ensure a comprehensive review of literature related to surgical techniques and bone defects in the rat femur. The search aimed to capture *in vivo* studies published in English from January 1st, 2001 to January 1st, 2023. The search strategy was built around three key components: anatomical focus (rat femur), relevant bone defects, and associated surgical techniques. The search formula was developed based on primary studies, previous systematic reviews, and an analysis of relevant MeSH terms and keywords.

The relevance of search terms was validated using PubMed's PubReMiner tool for frequency analysis of keywords and the Yale MeSH Analyzer to check the appropriateness of MeSH terms. The finalized search formula was tested against a set of known studies. Additional searches were performed for gray literature using Google Scholar and relevant conference abstracts.

Search Formula: The search formula used across MEDLINE (PubMed) and Embase databases is presented below.

**Search Methodology**	
**MEDLINE (PubMed)**	((“Rats”[MeSH] OR “Rodentia”[MeSH] OR “Rodents”[tiab] OR “Rattus”[tiab] OR "rat"[tiab] OR "rats"[tiab] OR "rodent"[tiab] OR "rodents"[tiab] OR “Sprague-Dawley”[tiab] OR "Wistar"[tiab] OR "Rattus norvegicus"[tiab] OR “murinae”[tiab])) AND ((“Femur”[MeSH] OR “Bone Defect*”[tiab] OR “Fractures, Bone”[MeSH] OR "bone defect"[tiab] OR "femoral defect"[tiab] OR "femur defect"[tiab] OR "bone fracture"[tiab] OR "femur"[tiab] OR "femoral"[tiab] OR "cortical bone"[tiab] OR "diaphysis"[tiab] OR "metaphysis"[tiab] OR "trabecular bone"[tiab] OR "femoral fracture"[tiab] OR "segmental bone defect"[tiab] OR "segmental defect"[tiab] OR "segmental femur"[tiab] OR "osteotomy"[tiab] OR "ostectomy"[tiab] OR “critical-sized defect”[tiab] OR "non-union"[tiab])) AND ((“Bone Regeneration”[MeSH] OR “Fracture Healing”[MeSH] OR "osteogenesis"[tiab] OR "bone healing"[tiab] OR "callus formation"[tiab] OR "bone regeneration"[tiab] OR "osteoconduction"[tiab] OR "osteopromotion"[tiab] OR "bone remodeling"[tiab] OR "bone repair"[tiab] OR "regenerative medicine"[tiab] OR "bone grafting"[tiab] OR "biomaterial"[tiab] OR "bone scaffold"[tiab] OR "biocompatibility"[tiab] OR "bone surgery"[tiab] OR "implant"[tiab] OR "plate fixation"[tiab] OR "osteosynthesis"[tiab] OR "internal fixation"[tiab] OR "external fixation"[tiab])) AND (("in vivo"[tiab] OR “animal model”[tiab] OR "animal models"[tiab] OR “experimental model”[tiab] OR “preclinical study”[tiab] OR "preclinical research"[tiab] OR "preclinical studies"[tiab]))
**Embase**	(('rat'/exp OR 'rodentia'/exp OR 'rodent':ti,ab OR 'rattus':ti,ab OR 'rat':ti,ab OR 'rats':ti,ab OR 'rodent':ti,ab OR 'rodents':ti,ab OR 'sprague dawley':ti,ab OR 'wistar':ti,ab OR 'rattus norvegicus':ti,ab OR 'murinae':ti,ab)<br>AND

	('femur'/exp OR 'bone defect'/exp OR 'bone defect*':ti,ab OR 'fracture'/exp OR 'bone defect':ti,ab OR 'femoral defect':ti,ab OR 'femur defect':ti,ab OR 'bone fracture':ti,ab OR 'femur':ti,ab OR 'femoral':ti,ab OR 'cortical bone':ti,ab OR 'diaphysis':ti,ab OR 'metaphysis':ti,ab OR 'trabecular bone':ti,ab OR 'femoral fracture':ti,ab OR 'segmental bone defect':ti,ab OR 'segmental defect':ti,ab OR 'segmental femur':ti,ab OR 'osteotomy':ti,ab OR 'ostectomy':ti,ab OR 'critical sized defect':ti,ab OR 'non union':ti,ab)<br>AND<br>('bone regeneration'/exp OR 'fracture healing'/exp OR 'osteogenesis':ti,ab OR 'bone healing':ti,ab OR 'callus formation':ti,ab OR 'bone regeneration':ti,ab OR 'osteoconduction':ti,ab OR 'osteopromotion':ti,ab OR 'bone remodeling':ti,ab OR 'bone repair':ti,ab OR 'regenerative medicine':ti,ab OR 'bone grafting':ti,ab OR 'biomaterial':ti,ab OR 'bone scaffold':ti,ab OR 'biocompatibility':ti,ab OR 'bone surgery':ti,ab OR 'implant':ti,ab OR 'plate fixation':ti,ab OR 'osteosynthesis':ti,ab OR 'internal fixation':ti,ab OR 'external fixation':ti,ab)<br>AND<br>('in vivo':ti,ab OR 'animal model':ti,ab OR 'animal models':ti,ab OR 'experimental model':ti,ab OR 'preclinical study':ti,ab OR 'preclinical research':ti,ab OR 'preclinical studies':ti,ab))
